# Correction: Impact of disease stage and age at Parkinson’s onset on patients’ primary concerns: Insights for targeted management

**DOI:** 10.1371/journal.pone.0252988

**Published:** 2021-06-04

**Authors:** Roongroj Bhidayasiri, Thanatat Boonmongkol, Yuwadee Thongchuam, Saisamorn Phumphid, Nitinan Kantachadvanich, Pattamon Panyakaew, Priya Jagota, Rachaneewan Plengsri, Marisa Chokpatcharavate, Onanong Phokaewvarangkul

In the Introduction, there is an error in the last sentence of the second paragraph. The correct sentence is: A larger survey from Thailand found significant knowledge gaps amongst PD patients across three domains: diagnosis, therapeutic options, and disease course, suggesting the need for education.

There are errors in Table 1. Numerous values in the Results column are incorrect. Please see the correct Table 1 here.

**Table 1:** Demographics of the 222 PD patients who completed the Parkinson’s disease patients’ concerns survey.

Item	Results
Age	67.69 ± 10.84
Male gender	125 (56.3%)
Disease duration	10.40 ± 6.58
Presenting symptoms at the time of diagnosis:	
■ Bradykinesia	127 (61.7%)
■ Tremor	108 (48.6%)
■ Rigidity	93 (41.9%)
■ Gait difficulty	87 (39.2%)
Mean HY Score (all patients)	2.76 ± 0.91
Number at each HY stage:	
■ Stage 1	6 (2.7%)
■ Stage 2	97 (43.7%)
■ Stage 3	73 (32.9%)
■ Stage 4	36 (16.2%)
■ Stage 5	10 (4.5%)
Presence of postural instability	119 (53.6%)
Presence of caregivers	112 (50.5%)
Categorical data are reported as number and percentage; continuous data are reported as mean ± standard deviation. HY: Hoehn & Yahr stage	

There is an error in Table 5. In the second Overall severity column, the result for the symptom “Turning in bed” is missing. Please see the correct Table 5 here.

**Table 5:** Comparison of the most commonly concerning symptoms between young-onset and typical-onset PD patients on (a) motor symptoms, (b) non-motor symptoms, (c) symptom fluctuations, (d) adverse events, and (e) care in the advanced stage (n = 222).

Young-onset PD		Typical-onset PD	
Symptom (%)	Overall severity	Symptom (%)	Overall severity
** a. Motor symptoms**			
Freezing of gait (46.3%)	5.37 ± 3.40	Getting out of bed (39.5%) Problems with walking and/or balance (39.5%)	5.04 ± 3.10 5.31 ± 2.94
Problems with walking and/or balance (44.4%)	5.87 ± 2.91	Freezing of gait (33.5%)	4.77 ± 3.39
Difficulty speaking (42.6%)	5.65 ± 2.99	Turning in bed (29.3%)	+ 3.33
** b. Non-motor symptoms**			
Constipation (48.1%)	5.67 ± 3.19	Constipation (38.9%)	4.99 ± 3.46
Fatigue (31.5%)	4.81 ± 3.03	Fatigue (31.1%)	4.26 ± 2.97
Daytime sleepiness (27.8%) Anxiety and/or panic attacks (27.8%)	4.22 ± 2.75 4.81 ± 2.50	Urinary problems (29.9%)	4.25 ± 3.22
** c. Symptom fluctuations**			
Slow movement (57.4%)	6.46 ± 2.62	Slow movement (36.5%)	4.80 ± 3.10
Difficulty performing fine finger movements (53.7%)	6.26 ± 2.72	Muscle cramping (31.7%) Pain and/or aching (31.7%)	4.41 ± 3.13 4.66 ± 2.89
Any stiffness (50.0%) Muscle cramping (50.0%)	5.76 ± 3.05 5.96 ± 2.75	Any stiffness (29.3%)	4.23 ± 3.13
** d. Adverse events**			
Gastrointestinal symptoms (37%)	4.72 ± 3.40	Gastrointestinal symptoms (29.9%)	4.47 ± 3.17
Urinary symptoms (31.5%)	4.35 ± 3.41	Urinary symptoms (21.6%)	3.84 ± 3.23
General symptoms (16.7%) Neuropsychiatric symptoms (16.7%)	3.44 ± 2.85 3.33 ± 2.91	Neuropsychiatric symptoms (16.8%)	2.95 ± 2.98
** e. Care in the advanced stage**			
Marked decline in physical ability (53.7%)	6.63 ± 2.84	Marked decline in physical ability (35.9%)	5.01 ± 3.24
Bedridden/wheelchair bound (44.4%)	5.26 ± 4.05	Bedridden/wheelchair bound (31.7%)	4.18 ± 3.78
Difficulty swallowing (42.6%)	4.67 ± 3.61	Cognitive difficulties (28.1%)	3.95 ± 3.29
Severity scores are reported as mean ± standard deviation.			
