# Supplementary material for: Impact of disease stage and age at Parkinson’s onset on patients’ primary concerns: Insights for targeted management
Source: PLoS One. 2020 Dec 2;15(12):e0243051. doi: 10.1371/journal.pone.0243051 (PMC7710032; doi:10.1371/journal.pone.0243051)
Supplement: S1 Table — (DOCX) [file pone.0243051.s001.docx]

**S1 Table.** Survey on Parkinson’s Disease Patients’ Concerns (PDPC Survey).

**Parkinson’s Disease Patients’ Concerns Survey**

** Please rate your level of concern about the following symptoms. Please mark a cross in the relevant box that best reflects your opinion.

| Not happy at all | | | | |  | |  | |  | |  | |  | | Very happy | | | | | | |  |  |
| --- | --- | --- | --- | --- | --- | --- | --- | --- | --- | --- | --- | --- | --- | --- | --- | --- | --- | --- | --- | --- | --- | --- | --- |
| Happiness | 1 | | 2 | | 3 | | 4 | | 5 | | 6 | | 7 | | 8 | | 9 | | 10 | |  |  |  |
| 1. Level of happiness | |  | |  | |  | |  | |  | |  | |  | |  | |  | |  |  |  |  |
|  | | | | |  | |  | |  | |  | |  | |  | |  | | | | | |  |
| Not concerned | | | | |  | |  | |  | |  | |  | |  | | Most concerned | | | | | |  |
| Concerns about motor symptoms | 1 | | 2 | | 3 | | 4 | | 5 | | 6 | | 7 | | 8 | | 9 | | 10 | |  |  |  |
| 1. Difficulty speaking | |  | |  | |  | |  | |  | |  | |  | |  | |  | |  |  |  |  |
| 1. Saliva and drooling | |  | |  | |  | |  | |  | |  | |  | |  | |  | |  |  |  |  |
| 1. Difficulty chewing and swallowing | |  | |  | |  | |  | |  | |  | |  | |  | |  | |  |  |  |  |
| 1. Eating tasks | |  | |  | |  | |  | |  | |  | |  | |  | |  | |  |  |  |  |
| 1. Dressing | |  | |  | |  | |  | |  | |  | |  | |  | |  | |  |  |  |  |
| 1. Washing and bathing | |  | |  | |  | |  | |  | |  | |  | |  | |  | |  |  |  |  |
| 1. Social activities | |  | |  | |  | |  | |  | |  | |  | |  | |  | |  |  |  |  |
| 1. Shaking | |  | |  | |  | |  | |  | |  | |  | |  | |  | |  |  |  |  |
| 1. Turning in bed | |  | |  | |  | |  | |  | |  | |  | |  | |  | |  |  |  |  |
| 1. Getting out of bed | |  | |  | |  | |  | |  | |  | |  | |  | |  | |  |  |  |  |
| 1. Problems with walking and/or balance | |  | |  | |  | |  | |  | |  | |  | |  | |  | |  |  |  |  |
| 1. Freezing of gait (temporary inability to move) | |  | |  | |  | |  | |  | |  | |  | |  | |  | |  |  |  |  |
|  | | | | | |  | |  | |  | |  | |  | |  | |  | | | | | |
| Not concerned | | | | | |  | |  | |  | |  | |  | |  | | Most concerned | | | | | |
| Concerns about non-motor symptoms | | 1 | | 2 | | 3 | | 4 | | 5 | | 6 | | 7 | | 8 | | 9 | | 10 |  |  |  |
| 1. Cognitive difficulties | |  | |  | |  | |  | |  | |  | |  | |  | |  | |  |  |  |  |
| 1. Hallucinations and delusions | |  | |  | |  | |  | |  | |  | |  | |  | |  | |  |  |  |  |
| 1. Low and/or depressed mood | |  | |  | |  | |  | |  | |  | |  | |  | |  | |  |  |  |  |
| 1. Anxiety and/or panic attacks | |  | |  | |  | |  | |  | |  | |  | |  | |  | |  |  |  |  |
| 1. Lack of interest or enthusiasm | |  | |  | |  | |  | |  | |  | |  | |  | |  | |  |  |  |  |
| 1. Lack of self-control (e.g. craving for, or strong impulse to take, medications in the absence of symptoms) | |  | |  | |  | |  | |  | |  | |  | |  | |  | |  |  |  |  |
| 1. Insomnia | |  | |  | |  | |  | |  | |  | |  | |  | |  | |  |  |  |  |
| 1. Daytime sleepiness | |  | |  | |  | |  | |  | |  | |  | |  | |  | |  |  |  |  |
| 1. Urinary problems | |  | |  | |  | |  | |  | |  | |  | |  | |  | |  |  |  |  |
| 1. Pain and other sensations | |  | |  | |  | |  | |  | |  | |  | |  | |  | |  |  |  |  |
| 1. Constipation | |  | |  | |  | |  | |  | |  | |  | |  | |  | |  |  |  |  |
| 1. Light headedness when standing | |  | |  | |  | |  | |  | |  | |  | |  | |  | |  |  |  |  |
| 1. Fatigue | |  | |  | |  | |  | |  | |  | |  | |  | |  | |  |  |  |  |
|  | | | | | |  | |  | |  | |  | |  | |  | |  | | | | | |
| Not concerned | | | | | |  | |  | |  | |  | |  | |  | | Most concerned | | | | | |
| Concerns about symptom fluctuations | | 1 | | 2 | | 3 | | 4 | | 5 | | 6 | | 7 | | 8 | | 9 | | 10 |  |  |  |
| 1. Shaking | |  | |  | |  | |  | |  | |  | |  | |  | |  | |  |  |  |  |
| 1. Anxiety and/or panic attacks | |  | |  | |  | |  | |  | |  | |  | |  | |  | |  |  |  |  |
| 1. Mood changes | |  | |  | |  | |  | |  | |  | |  | |  | |  | |  |  |  |  |
| 1. Slow movement | |  | |  | |  | |  | |  | |  | |  | |  | |  | |  |  |  |  |
| 1. Difficulty performing fine finger movements | |  | |  | |  | |  | |  | |  | |  | |  | |  | |  |  |  |  |
| 1. Any stiffness | |  | |  | |  | |  | |  | |  | |  | |  | |  | |  |  |  |  |
| 1. Muscle cramping | |  | |  | |  | |  | |  | |  | |  | |  | |  | |  |  |  |  |
| 1. Pain and/or aching | |  | |  | |  | |  | |  | |  | |  | |  | |  | |  |  |  |  |
| 1. Drug-induced dyskinesia | |  | |  | |  | |  | |  | |  | |  | |  | |  | |  |  |  |  |
|  | | | | | |  | |  | |  | |  | |  | |  | |  | | | | | |
| Not concerned | | | | | |  | |  | |  | |  | |  | |  | | Most concerned | | | | | |
| Concerns about adverse events | | 1 | | 2 | | 3 | | 4 | | 5 | | 6 | | 7 | | 8 | | 9 | | 10 |  |  |  |
| 1. General symptoms | |  | |  | |  | |  | |  | |  | |  | |  | |  | |  |  |  |  |
| 1. Cardiovascular symptoms | |  | |  | |  | |  | |  | |  | |  | |  | |  | |  |  |  |  |
| 1. Gastrointestinal symptoms | |  | |  | |  | |  | |  | |  | |  | |  | |  | |  |  |  |  |
| 1. Urinary symptoms | |  | |  | |  | |  | |  | |  | |  | |  | |  | |  |  |  |  |
| 1. Neuropsychiatric symptoms | |  | |  | |  | |  | |  | |  | |  | |  | |  | |  |  |  |  |
| 1. Dermatologic symptoms | |  | |  | |  | |  | |  | |  | |  | |  | |  | |  |  |  |  |
|  | | | | | |  | |  | |  | |  | |  | |  | |  | | | | | |
| Not concerned | | | | | |  | |  | |  | |  | |  | |  | | Most concerned | | | | | |
| Concerns about care in the advanced stage of PD | | 1 | | 2 | | 3 | | 4 | | 5 | | 6 | | 7 | | 8 | | 9 | | 10 |  |  |  |
| 1. Difficulty swallowing | |  | |  | |  | |  | |  | |  | |  | |  | |  | |  |  |  |  |
| 1. Recurrent infections | |  | |  | |  | |  | |  | |  | |  | |  | |  | |  |  |  |  |
| 1. Marked decline in physical ability | |  | |  | |  | |  | |  | |  | |  | |  | |  | |  |  |  |  |
| 1. Aspiration pneumonia | |  | |  | |  | |  | |  | |  | |  | |  | |  | |  |  |  |  |
| 1. Cognitive difficulties | |  | |  | |  | |  | |  | |  | |  | |  | |  | |  |  |  |  |
| 1. Weight loss | |  | |  | |  | |  | |  | |  | |  | |  | |  | |  |  |  |  |
| 1. Bedridden / wheelchair bound | |  | |  | |  | |  | |  | |  | |  | |  | |  | |  |  |  |  |

| Concerns about the need for assistive devices | Need | No need |
| --- | --- | --- |
| 1. Assistive device for patient |  |  |
| - 1. Walking stick |  |  |
| - 1. Laser-guided walking stick providing visual cues |  |  |
| - 1. Walker |  |  |
| - 1. Wheelchair |  |  |
| - 1. Electric adjustable bed |  |  |
| - 1. Wearables for fall detection |  |  |
| - 1. Tremor suppression spoon |  |  |
| - 1. Tremor suppression gloves |  |  |
| 1. Home adaptation devices |  |  |
| - 1. Bed rails |  |  |
| - 1. Home rails |  |  |
| - 1. Bed support rails |  |  |
| - 1. Anti-slip mat |  |  |
| - 1. Cushioned fall mat |  |  |
| - 1. Horizontal lines on the floor as visual cues |  |  |
| - 1. Button-up device |  |  |
| - 1. Shower chair |  |  |
| - 1. Commode |  |  |
| - 1. Anti-choking cup |  |  |
| - 1. Fall alarm |  |  |
| - 1. Electric home ladder |  |  |
| - 1. Suction device |  |  |
