# Supplementary material for: Impact of disease stage and age at Parkinson’s onset on patients’ primary concerns: Insights for targeted management
Source: PLoS One. 2020 Dec 2;15(12):e0243051. doi: 10.1371/journal.pone.0243051 (PMC7710032; doi:10.1371/journal.pone.0243051)
Supplement: S2 Table — (DOCX) [file pone.0243051.s002.docx]

**S2 Table. Comparison of the characteristics and PDPC survey scores of patients with caregivers and those without.**

| Items | No caregivers (n=110) | Caregivers (n=112) | *p-*value | *Effect size* |
| --- | --- | --- | --- | --- |
| Age | 63.98 ± 9.91 | 71.34 ± 10.51 | <0.001* | 0.72 |
| Disease duration | 9.43 ± 6.18 | 11.34 ± 6.85 | 0.031* | 0.29 |
| H&Y stage | 2.25 ± 0.61 | 3.27 ± 0.88 | <0.001* | 1.34 |
| 1. Level of happiness | 6.47 ± 2.26 | 6.36 ± 2.22 | 0.701 | 0.05 |
| 1. Difficulty speaking | 3.98 ± 3.17 | 4.54 ± 3.26 | 0.200 | 0.17 |
| 1. Saliva and drooling | 3.03 ± 2.95 | 3.63 ± 3.29 | 0.15 | 0.19 |
| 1. Difficulty chewing swallowing | 2.52 ± 2.72 | 3.67 ± 3.17 | 0.004* | 0.39 |
| 1. Eating tasks | 2.40 ± 2.77 | 3.22 ± 3.02 | 0.036* | 0.28 |
| 1. Dressing | 2.61 ± 2.75 | 3.57 ± 3.12 | 0.015* | 0.33 |
| 1. Washing and bathing | 2.27 ± 2.57 | 3.09 ± 2.89 | 0.027* | 0.30 |
| 1. Social activities | 3.17 ± 2.92 | 4.22 ± 3.21 | 0.012* | 0.34 |
| 1. Shaking | 3.79 ± 2.97 | 4.04 ± 3.08 | 0.548 | 0.08 |
| 1. Turning in bed | 3.75 ± 3.29 | 4.88 ± 3.31 | 0.011* | 0.34 |
| 1. Getting out of bed | 4.38 ± 3.01 | 5.80 ± 2.97 | <0.001* | 0.47 |
| 1. Problems with walking and/or balance | 4.75 ± 2.95 | 6.13 ± 2.76 | <0.001* | 0.48 |
| 1. Freezing of gait (temporary inability to move) | 4.18 ± 3.40 | 5.64 ± 3.23 | 0.001* | 0.44 |
| 1. Cognitive difficulties | 3.25 ± 2.98 | 4.04 ± 3.00 | 0.048* | 0.26 |
| 1. Hallucinations and delusions | 1.83 ± 2.26 | 2.81 ± 3.02 | 0.009* | 0.37 |
| 1. Low and/or depressed mood | 2.62 ± 2.63 | 3.29 ± 2.87 | 0.069 | 0.24 |
| 1. Anxiety and/or panic attacks | 3.23 ± 2.54 | 4.03 ± 2.92 | 0.03* | 0.29 |
| 1. Lack of interest or enthusiasm | 3.28 ± 2.90 | 3.78 ± 2.76 | 0.194 | 0.18 |
| 1. Lack of self-control (e.g. craving for, or strong impulse to take, medications in the absence of symptoms) | 2.83 ± 2.79 | 3.49 ± 2.88 | 0.082 | 0.23 |
| 1. Insomnia | 3.57 ± 2.91 | 4.53 ± 3.22 | 0.021* | 0.31 |
| 1. Daytime sleepiness | 3.84 ± 2.86 | 4.56 ± 2.89 | 0.061 | 0.25 |
| 1. Urinary problems | 3.81 ± 3.08 | 4.48 ± 3.19 | 0.111 | 0.21 |
| 1. Pain and other sensations | 3.45 ± 2.93 | 3.80 ± 3.07 | 0.387 | 0.12 |
| 1. Constipation | 4.75 ± 3.29 | 5.56 ± 3.47 | 0.076 | 0.24 |
| 1. Light headedness when standing | 2.99 ± 2.75 | 3.64 ± 2.84 | 0.084 | 0.23 |
| 1. Fatigue | 3.94 ± 3.06 | 4.85 ± 2.85 | 0.022* | 0.31 |
| 1. Shaking | 3.83 ± 3.41 | 4.05 ± 3.00 | 0.600 | 0.07 |
| 1. Anxiety and/or panic attacks | 3.25 ± 3.13 | 3.58 ± 3.10 | 0.437 | 0.11 |
| 1. Mood changes | 3.22 ± 3.08 | 3.52 ± 2.97 | 0.462 | 0.10 |
| 1. Slow movement | 4.98 ± 3.18 | 5.43 ± 2.94 | 0.278 | 0.15 |
| 1. Difficulty performing fine finger movements | 4.81 ± 3.18 | 4.88 ± 3.05 | 0.858 | 0.02 |
| 1. Any stiffness | 4.38 ± 3.23 | 4.84 ± 3.11 | 0.283 | 0.15 |
| 1. Muscle cramping | 4.58 ± 3.13 | 5.02 ± 3.09 | 0.297 | 0.14 |
| 1. Pain and/or aching | 4.43 ± 3.05 | 5.26 ± 2.83 | 0.036* | 0.28 |
| 1. Drug-induced dyskinesia | 3.23 ± 3.16 | 2.64 ± 2.68 | 0.139 | 0.20 |
| 1. General symptoms | 2.84 ± 2.91 | 3.09 ± 2.75 | 0.507 | 0.09 |
| 1. Cardiovascular symptoms | 2.56 ± 2.76 | 2.99 ± 2.74 | 0.248 | 0.16 |
| 1. Gastrointestinal symptoms | 4.35 ± 3.22 | 4.68 ± 3.23 | 0.443 | 0.10 |
| 1. Urinary symptoms | 3.63 ± 3.17 | 4.29 ± 3.34 | 0.134 | 0.20 |
| 1. Neuropsychiatric symptoms | 2.76 ± 2.60 | 3.33 ± 3.26 | 0.153 | 0.19 |
| 1. Dermatologic symptoms | 2.32 ± 2.66 | 2.88 ± 2.94 | 0.134 | 0.20 |
| 1. Difficulty swallowing | 3.73 ± 3.42 | 4.54 ± 3.29 | 0.071 | 0.24 |
| 1. Recurrent infections | 2.82 ± 3.18 | 3.12 ± 3.17 | 0.486 | 0.09 |
| 1. Marked decline in physical ability | 5.18 ± 3.27 | 5.66 ± 3.16 | 0.268 | 0.15 |
| 1. Aspiration pneumonia | 2.54 ± 2.99 | 2.91 ± 3.31 | 0.378 | 0.11 |
| 1. Cognitive difficulties | 3.99 ± 3.22 | 4.44 ± 3.37 | 0.314 | 0.14 |
| 1. Weight loss | 3.04 ± 3.08 | 3.05 ± 2.84 | 0.965 | 0.003 |
| 1. Bedridden / wheelchair bound | 4.19 ± 3.95 | 4.74 ± 3.80 | 0.291 | 0.14 |
| All statistics were performed using an unpaired t-test for continuous units. A *p*-value less than 0.05 was considered statistically significant. | | | | |
