# Supplementary material for: Impact of disease stage and age at Parkinson’s onset on patients’ primary concerns: Insights for targeted management
Source: PLoS One. 2020 Dec 2;15(12):e0243051. doi: 10.1371/journal.pone.0243051 (PMC7710032; doi:10.1371/journal.pone.0243051)
Supplement: S3 Table — (DOCX) [file pone.0243051.s003.docx]

**S3 Table.** Patients’ concerns on motor symptoms, non-motor symptoms, and symptom fluctuations: comparison of patients with young-onset and typical onset PD.

| Items | Severity of concern | Young-onset PD (N=54) | Typical onset PD (N=168) | *p*-value |
| --- | --- | --- | --- | --- |
| **Concern about motor symptoms** | | | | |
| Difficulty speaking | no concern | 2 (3.7%) | 40 (23.4%) | 0.003* |
|  | Some concern | 29 (53.7%) | 84 (50.3%) |  |
|  | Most concern | 23 (42.6%) | 44 (26.3%) |  |
| Saliva and drooling | no concern | 9 (16.7%) | 52 (30.5%) | 0.23 |
|  | Some concern | 32 (59.3%) | 82 (49.1%) |  |
|  | Most concern | 13 (24.1%) | 34 (20.4%) |  |
| Difficulty chewing swallowing | no concern | 13 (24.1%) | 51 (29.9%) | 0.22 |
|  | Some concern | 31 (57.4%) | 92 (55.1%) |  |
|  | Most concern | 10 (18.5%) | 25 (15%) |  |
| Eating tasks | no concern | 12 (22.2%) | 58 (34.1%) | 0.003* |
|  | Some concern | 33 (61.1%) | 92 (55.1%) |  |
|  | Most concern | 9 (16.7%) | 18 (10.8%) |  |
| Dressing | no concern | 11 (20.4%) | 53 (31.1%) | 0.13 |
|  | Some concern | 34 (62.9%) | 92 (55.1%) |  |
|  | Most concern | 9 (16.7%) | 23 (13.8%) |  |
| Washing and bathing | no concern | 15 (27.8%) | 51 (29.9%) | 0.04* |
|  | Some concern | 36 (66.7%) | 99 (59.3%) |  |
|  | Most concern | 3 (5.6%) | 18 (10.8%) |  |
| Social activities | no concern | 7 (13%) | 39 (22.8%) | 0.15 |
|  | Some concern | 35 (54.8%) | 98 (58.6%) |  |
|  | Most concern | 12 (22.2%) | 31 (18.6%) |  |
| Shaking | no concern | 9 (16.7%) | 30 (17.4%) | 0.06 |
|  | Some concern | 25 (46.3%) | 104 (62.2%) |  |
|  | Most concern | 20 (37%) | 34 (20.4%) |  |
| Turning in bed | no concern | 9 (16.7%) | 30 (17.4%) | 0.84 |
|  | Some concern | 26 (48.1%) | 89 (53.3%) |  |
|  | Most concern | 19 (35.2%) | 49 (29.3%) |  |
| Getting out of bed | no concern | 5 (9.3%) | 16 (9%) | 0.66 |
|  | Some concern | 27 (50%) | 86 (51.5%) |  |
|  | Most concern | 22 (40.7%) | 66 (39.5%) |  |
| Problems with walking and/or balance | no concern | 2 (3.7%) | 8 (4.2%) | 0.35 |
|  | Some concern | 28 (51.8%) | 94 (56.2%) |  |
|  | Most concern | 24 (44.4%) | 66 (39.5%) |  |
| Freezing of gait (temporary inability to move) | no concern | 7 (13%) | 26 (15.1%) | 0.38 |
|  | Some concern | 22 (40.7%) | 86 (51.4%) |  |
|  | Most concern | 25 (46.3%) | 56 (33.5%) |  |
| **Concern about non-motor symptoms** | | | | |
| Cognitive difficulties | no concern | 8 (14.8%) | 39 (22.8%) | 0.19 |
|  | Some concern | 34 (62.9%) | 93 (55.7%) |  |
|  | Most concern | 12 (22.2%) | 36 (21.6%) |  |
| Hallucinations and delusions | no concern | 19 (35.2%) | 76 (44.9%) | 0.62 |
|  | Some concern | 27 (50%) | 72 (43.1%) |  |
|  | Most concern | 8 (14.8%) | 20 (12%) |  |
| Low and/or depressed mood | no concern | 6 (11.1%) | 57 (33.5%) | 0.01* |
|  | Some concern | 40 (64.1%) | 92 (55.1%) |  |
|  | Most concern | 8 (14.8%) | 19 (11.4%) |  |
| Anxiety and/or panic attacks | no concern | 3 (5.6%) | 37 (21.6%) | <0.001* |
|  | Some concern | 36 (66.6%) | 105 (62.8%) |  |
|  | Most concern | 15 (27.8%) | 26 (15.6%) |  |
| Lack of interest or enthusiasm | no concern | 8 (14.8%) | 39 (22.8%) | 0.07 |
|  | Some concern | 35 (64.8%) | 103 (61.6%) |  |
|  | Most concern | 11 (20.4%) | 26 (15.6%) |  |
| Lack of self-control (e.g. craving for, or strong impulse to take, medications in the absence of symptoms) | no concern | 12 (22.2%) | 46 (26.9%) | 0.20 |
|  | Some concern | 31 (57.4%) | 96 (57.5%) |  |
|  | Most concern | 11 (20.4%) | 26 (15.6%) |  |
| Insomnia | no concern | 12 (22.2%) | 31 (18%) | 0.29 |
|  | Some concern | 29 (53.7%) | 90 (53.9%) |  |
|  | Most concern | 13 (24.1%) | 47 (28.1%) |  |
| Daytime sleepiness | no concern | 7 (13%) | 23 (13.3%) | 0.99 |
|  | Some concern | 32 (59.3%) | 101 (60.4%) |  |
|  | Most concern | 15 (27.8%) | 44 (26.3%) |  |
| Urinary problems | no concern | 11 (20.4%) | 27 (15.6%) | 0.25 |
|  | Some concern | 32 (59.3%) | 91 (54.5%) |  |
|  | Most concern | 11 (20.4%) | 50 (29.9%) |  |
| Pain and other sensations | no concern | 5 (9.3%) | 34 (19.8%) | 0.07 |
|  | Some concern | 36 (64.8%) | 102 (61.1%) |  |
|  | Most concern | 14 (25.9%) | 32 (19.2%) |  |
| Constipation | no concern | 6 (11.1%) | 24 (13.8%) | 0.32 |
|  | Some concern | 22 (40.7%) | 79 (47.3%) |  |
|  | Most concern | 26 (48.1%) | 65 (38.9%) |  |
| Light headedness when standing | no concern | 12 (22.2%) | 33 (19.2%) | 0.92 |
|  | Some concern | 33 (61.1%) | 106 (63.4%) |  |
|  | Most concern | 9 (16.7%) | 29 (17.4%) |  |
| Fatigue | no concern | 4 (7.4%) | 20 (11.4%) | 0.06 |
|  | Some concern | 33 (61.1%) | 96 (57.5%) |  |
|  | Most concern | 17 (31.5%) | 52 (31.1%) |  |
| **Concern about symptoms fluctuations** | | | | |
| Shaking | no concern | 8 (14.8%) | 33 (19.8%) | 0.005* |
|  | Some concern | 26 (47.2%) | 98 (58.7%) |  |
|  | Most concern | 20 (37%) | 36 (21.6%) |  |
| Anxiety and/or panic attacks | no concern | 8 (14.8%) | 44 (26.3%) | 0.02* |
|  | Some concern | 29 (53.7%) | 96 (57.5%) |  |
|  | Most concern | 17 (31.5%) | 27 (16.2%) |  |
| Mood changes | no concern | 9 (16.7%) | 42 (25.1%) | 0.003* |
|  | Some concern | 28 (50%) | 96 (56.9%) |  |
|  | Most concern | 18 (33.3%) | 30 (18%) |  |
| Slow movement | no concern | 1 (1.9%) | 16 (9.6%) | 0.007* |
|  | Some concern | 22 (40.8%) | 90 (53.9%) |  |
|  | Most concern | 31 (57.4%) | 61 (36.5%) |  |
| Difficulty performing fine finger movements | no concern | 0 | 19 (11.4%) | 0.001* |
|  | Some concern | 25 (46.3%) | 100 (59.8%) |  |
|  | Most concern | 29 (53.7%) | 48 (28.7%) |  |
| Any stiffness | no concern | 1 (1.9%) | 24 (14.4%) | 0.01* |
|  | Some concern | 27 (48.2%) | 94 (56.3%) |  |
|  | Most concern | 27 (50%) | 49 (29.3%) |  |
| Muscle cramping | no concern | 0 (0%) | 24 (14.4%) | 0.007* |
|  | Some concern | 27 (50%) | 90 (53.9%) |  |
|  | Most concern | 27 (50%) | 53 (31.7%) |  |
| Pain and/or aching | no concern | 2 (3.7%) | 13 (7.8%) | 0.63 |
|  | Some concern | 31 (57.4%) | 101 (60.4%) |  |
|  | Most concern | 21 (38.9%) | 53 (31.7%) |  |
| Drug-induced dyskinesia | no concern | 12 (22.2%) | 55 (32.9%) | <0.001* |
|  | Some concern | 24 (44.4%) | 97 (58.1%) |  |
|  | Most concern | 18 (33.3%) | 15 (9%) |  |
| All statistics were performed with Chi-square test for categorical units. *P*-value less than 0.05 was considered statistically significant. | | | | |
