# Supplementary material for: Impact of disease stage and age at Parkinson’s onset on patients’ primary concerns: Insights for targeted management
Source: PLoS One. 2020 Dec 2;15(12):e0243051. doi: 10.1371/journal.pone.0243051 (PMC7710032; doi:10.1371/journal.pone.0243051)
Supplement: S1 Data — (DOCX) [file pone.0243051.s004.docx]

**S1 Data.** Development and content validation of the Parkinson’s Disease Patients’ Concerns Survey (PDPC Survey).

1. **Content validity**

The Parkinson’s Disease Patients’ Concerns Survey (PDPC Survey) was developed by multidisciplinary care team at Chulalongkorn Centre of Excellence for Parkinson’s Disease and Related Disorders at Chulalongkorn Hospital, Bangkok, Thailand, to help identify patients’ concerns about their PD symptoms in the following categories: level of happiness, motor and non-motor symptoms, fluctuating motor symptoms, adverse events, palliative care, and the need for assistive devices.

A higher PDPC Survey score represents greater concern about that aspect of PD. Patients are asked to rate the severity of their concern on a scale of 0–10 where zero represents no concern and 10 represents severe concern.

The 50-item questionnaire was developed by two bilingual neurologists who specialise in movement disorders (KS, SR, RT, AL, ALW). Comprehension of each item was validated by five bilingual neurologists who specialise in movement disorders who checked for content validity using the Item Objective Congruence (IOC) index (Table 1). For each questionnaire item, the mean value on the IOC index was above 0.6, indicating positive content validation.

As a pilot trial, the survey was given to 30 literate PD patients and their caregivers to test whether they understood the meaning and the instructions. Following the trial, minor revisions were made to wording of the questionnaire. The PDPC Survey was translated into the Thai language by the Chalermprakiat Centre of Translation and Interpretation, Faculty of Arts, Chulalongkorn University. Comprehension of all items following translation was validated by two neurologists.

Backward translation into English was conducted by two neurologists who were not involved in the forward translation. Another neurologist compared the backward translation with the original English version. Given that the meaning of the backward translation is very similar to the original English version, the translated Thai version was accepted.

1. **Reliability (internal consistency)**

The reliability of PDPC Survey was assessed for Internal consistency by administering the test to 30 randomly selected PD patients who visited the Movement Disorders Outpatient Clinic of Chulalongkorn Centre of Excellence for Parkinson’s Disease and Related Disorders, Chulalongkorn University Hospital.

Internal consistency of the PDPC Survey was assessed with Cronbach’s alpha coefficient. These results indicated highly significant reliability of the PDPC Survey among our PD patients as follows:

- Overall PDPC Survey = 0.968 (r). (48 items, including subjective happiness rating: patients are asked to rate their overall happiness using a10-point scale where 1 is ‘very unhappy’ and 10 is ‘very happy’)
- Motor and non-motor section of the PDPC Survey = 0.946 (r). (25 items, based on the MDS–UPDRS parts I, II and III [1])
- Motor fluctuations section of the PDPC Survey = 0.922 (r). (9 items, based on WOQ-9 on [2], plus one item on dyskinesia)
- Adverse events section of the PDPC Survey = 0.839 (r). (6 items, based on Perez-Lloret et al. 2012 [3])
- Care in the advanced stage section of the PDPC Survey = 0.907 (r). (7 items, based on the UK National Health Service’s National End of Life Care Programme, 2013 [4])
- Need for assistive devices section (2 items). The items for this section were obtained from clinical interviews with representatives from Chulalongkorn Parkinsons’ Patient Support Group, literature review, and knowledge-based materials from various Parkinson’s disease foundations. Responses were based on their interest of these items. Therefore, validation of this section was not applicable.

**Table 1:** Item Objective Congruence (IOC) index scores for each item of the Parkinson’s Disease Patients’ Concerns Survey (PDPC Survey). Scores above 0.6 indicate positive content validation.

| Item | Participants | | | | | IOC index score |
| --- | --- | --- | --- | --- | --- | --- |
|  | KS | SR | RT | AL | ALW |  |
| Happiness level (1) |  |  |  |  |  |  |
| Presence of happiness | +1 | 0 | +1 | +1 | +1 | 0.8 |
| Motor section (12) |  |  |  |  |  |  |
| Difficulty speaking | 0 | +1 | +1 | 0 | +1 | 0.6 |
| Saliva and drooling | +1 | +1 | +1 | 0 | +1 | 0.8 |
| Difficulty chewing swallowing | +1 | 0 | +1 | +1 | +1 | 0.8 |
| Eating tasks | 0 | +1 | 0 | +1 | +1 | 0.6 |
| Dressing | +1 | +1 | +1 | 0 | +1 | 0.8 |
| Washing and bathing | +1 | +1 | +1 | +1 | 0 | 0.8 |
| Social activities | 0 | +1 | +1 | +1 | +1 | 0.8 |
| Shaking | +1 | 0 | +1 | +1 | +1 | 0.8 |
| Turning in bed | +1 | +1 | +1 | 0 | +1 | 0.8 |
| Getting out of bed | 0 | +1 | 0 | +1 | +1 | 0.6 |
| Problems with walking and/or balance | +1 | +1 | +1 | 0 | +1 | 0.8 |
| Freezing of gait (temporary inability to move) | +1 | +1 | +1 | +1 | 0 | 0.8 |
| Non-motor section (13) |  |  |  |  |  |  |
| Cognitive difficulties | +1 | +1 | +1 | +1 | +1 | 1.0 |
| Hallucination and delusions | +1 | 0 | +1 | +1 | +1 | 0.8 |
| Low and/or depressed mood | 0 | +1 | 0 | +1 | +1 | 0.6 |
| Anxiety and/or panic attacks | +1 | +1 | +1 | 0 | +1 | 0.8 |
| Lack of interest or enthusiasm | +1 | +1 | +1 | +1 | 0 | 0.8 |
| Lack of self-control (e.g. craving for, or strong impulse to take, medications in the absence of symptoms) | 0 | +1 | +1 | 0 | +1 | 0.6 |
| Insomnia | +1 | +1 | +1 | +1 | +1 | 1.0 |
| Daytime sleepiness | +1 | 0 | +1 | +1 | +1 | 0.8 |
| Urinary problems | +1 | +1 | +1 | +1 | +1 | 1.0 |
| Pain and other sensations | +1 | 0 | +1 | +1 | +1 | 0.8 |
| Constipation | 0 | +1 | 0 | +1 | +1 | 0.6 |
| Light headedness when standing | +1 | +1 | +1 | 0 | +1 | 0.8 |
| Fatigue | +1 | +1 | +1 | +1 | 0 | 0.8 |
| Symptom fluctuations section (9) |  |  |  |  |  |  |
| Shaking | 0 | +1 | +1 | 0 | +1 | 0.6 |
| Anxiety and/or panic attacks | +1 | +1 | +1 | 0 | +1 | 0.8 |
| Mood changes | 0 | +1 | +1 | +1 | +1 | 0.8 |
| Slow movement | +1 | 0 | +1 | +1 | +1 | 0.8 |
| Difficulty performing fine finger movements | 0 | +1 | 0 | +1 | +1 | 0.6 |
| Any stiffness | +1 | +1 | +1 | 0 | +1 | 0.8 |
| Muscle cramping | 0 | +1 | +1 | 0 | +1 | 0.6 |
| Pain and/or aching | +1 | 0 | +1 | +1 | +1 | 0.8 |
| Drug-induced dyskinesia | +1 | 0 | +1 | +1 | +1 | 0.8 |
| Adverse event section (6) |  |  |  |  |  |  |
| General symptoms | +1 | 0 | +1 | +1 | +1 | 0.8 |
| Cardiovascular symptoms | 0 | +1 | 0 | +1 | +1 | 0.6 |
| Gastrointestinal symptoms | +1 | +1 | +1 | 0 | +1 | 0.8 |
| Urinary symptoms | +1 | +1 | +1 | +1 | 0 | 0.8 |
| Neuropsychiatric symptoms | 0 | +1 | +1 | 0 | +1 | 0.6 |
| Dermatologic symptoms | +1 | +1 | +1 | 0 | +1 | 0.8 |
| Care in the advanced stage (7) |  |  |  |  |  |  |
| Difficulty swallowing | +1 | 0 | +1 | +1 | +1 | 0.8 |
| Recurrent infection | 0 | +1 | 0 | +1 | +1 | 0.6 |
| Marked decline in physical ability | +1 | +1 | +1 | 0 | +1 | 0.8 |
| Aspiration pneumonia | +1 | +1 | +1 | +1 | 0 | 0.8 |
| Cognitive difficulties | 0 | +1 | +1 | 0 | +1 | 0.6 |
| Weight loss | +1 | +1 | 0 | +1 | +1 | 0.8 |
| Bedridden / wheelchair bound | 0 | +1 | +1 | +1 | +1 | 0.8 |
| IOC: Item Objective Congruence | | | | | | |

**References**

[1]. Goetz CG, Fahn S, Martinez-Martin P*, et al.* Movement Disorder Society-sponsored revision of the Unified Parkinson's Disease Rating Scale (MDS-UPDRS): Process, format, and clinimetric testing plan. *Mov Disord*. 2007 **22:** 41-47.

[2]. Stacy M, Hauser R, Oertel W*, et al.* End-of-dose wearing off in Parkinson disease: a 9-question survey assessment. *Clin Neuropharmacol*. 2006 **29:** 312-321.

[3]. Perez-Lloret S, Rey MV, Fabre N*, et al.* Do Parkinson's disease patients disclose their adverse events spontaneously? *Eur J Clin Pharmacol*. 2012 **68:** 857-865.

[4]. UK National Health Service National End of Life Care Programme. 2017.
