# Supplementary material for: Impact of disease stage and age at Parkinson’s onset on patients’ primary concerns: Insights for targeted management
Source: PLoS One. 2020 Dec 2;15(12):e0243051. doi: 10.1371/journal.pone.0243051 (PMC7710032; doi:10.1371/journal.pone.0243051)
Supplement: Attachment — Submitted filename: ResponseReviewers-PONE-D-20-18129R1.docx [file pone.0243051.s006.docx]

12 November 2020

Mathias Toft, MD., PhD.

Academic Editor

*PLoS One*

Dear Prof. Toft,

Re: Manuscript # PONE-D-20-18129R1: Impact of disease stage and age at Parkinson’s onset on patients’ primary concerns: Insights for targeted management

Thank you very much for your letter of the 30^th^ of October 2020. We found the editor’s and reviewer’s comments very helpful and respond to them as follows:

**Reviewer #1:** Thank you, this is a much improved version of the manuscript.

*Response:* We would like to thank the reviewer for his/her suggestions that have improved our manuscript.

**Reviewer #2:** The authors have done a laudable job of improving this paper. I have only a few remaining comments:

1) Now that I understand “most concerning” I think the results (including abstract) might be more accurately labeled as “most commonly concerning” (meaning item where the most people rated it as highly concerning rather than “most concerning”, which I would like mean as highest ratings of concern.

*Response:* Thanks so much for this suggestion. We have revised the manuscript to use the term ‘most commonly concerning’ instead of ‘most concerning’ in the manuscript. Moreover, we have clarified the term ‘most commonly concerning’ with the definition suggested by the reviewer. It reads as follows:

(Page 9)

Results were reported in a form of most commonly concerning symptoms, referring to what the most patients rated these symptoms as highly concerning.

2) In Methods, it may be prudent to rename the first section “Survey development and partial validation” or “Survey development and steps of validation” and/or end section by saying that “other aspects of survey development and validation were not pursued” to make it clear to readers that this is not a fully validated or clinimetrically tested instrument.

*Response:* We have renamed the section as “Survey development and steps of validation” and also ended the section by stating that other aspects of survey development and validation were not performed as shown on pages 7 and 8.

3) In tables, I would eliminate the “Ranked 1^st^” “Ranked 2^nd^” …. The order of the items are clear. I think the “Ranked 1^st^” adds some confusion as it gives the impression that patients actually rank ordered their responses, which did not happen as I understand it.

*Response:* Tables 2, 4, and 5 were revised as suggested by the reviewer.

4) The results section ‘caregiver support’ should be renamed. Perhaps “concerns in advanced disease’ as it really does not assess caregiver support. Also, Table 3 should be similarly renamed or at minimum state items associated with presence of a caregiver rather than “predictors” which implies a temporal and causal association.

*Response:* Revision of a section name and revision of table 3 were performed as suggested by the reviewer.

5) I would change sentence on dementia in the Limitations section to something like: “As the majority of patients with PD will experience dementia, their exclusion in this study may limit the generalizability of results.

*Response:* As suggested by the reviewer, we have revised the sentence to be as follows:

(Page 34)

As the majority of PD patients will experience dementia, the exclusion of this patient group from the study may limit the generalisability of results.

I would like to confirm that all authors have read the manuscript; the paper has not been previously published, and is not under simultaneous consideration by another journal. There is also no ghost writing by anyone not named on the author list.

There is no conflict of interest on all authors and we will take full responsibility for the data, the analyses and interpretation, and the conduct of the research. We had full access to all of the data; and that we had the right to publish any and all data, separate and apart from the attitudes of the sponsor.

We would like to confirm that the manuscript has been reviewed by a native English speaker for style and grammatical accuracy. Thank you very much for consideration our manuscript for publication. Please let me know if there are any questions.

We are grateful to the editors and reviewers for the time and effort that they have put into helping us improve our manuscript.

Sincerely,

Roongroj Bhidayasiri

Corresponding author:

Roongroj Bhidayasiri, MD., FRCP., FRCPI.

Chulalongkorn Center of Excellence on Parkinson Disease and Related Disorders

Chulalongkorn University Hospital

1873 Rama 4 Road

Bangkok 10330

Thailand

Tel: +662-256-4000 ext. 70701

Fax: +662-256-4630

Email address: rbh@chulapd.org
